# Association between Plasma Homocysteine Levels and Neuronal Injury in HIV Infection

**DOI:** 10.1371/journal.pone.0158973

**Published:** 2016-07-21

**Authors:** Erika Ahlgren, Lars Hagberg, Dietmar Fuchs, Lars-Magnus Andersson, Staffan Nilsson, Henrik Zetterberg, Magnus Gisslén

**Affiliations:** 1 Department of Infectious Diseases, Institute of Biomedicine, Sahlgrenska Academy, University of Gothenburg, Gothenburg, Sweden; 2 Division of Biological Chemistry, Biocenter, Innsbruck Medical University, Innsbruck, Austria; 3 Mathematical Sciences, Chalmers University of Technology, Gothenburg, Sweden; 4 Department of Psychiatry and Neurochemistry, Institute of Neuroscience and Physiology, Sahlgrenska Academy, University of Gothenburg, Mölndal, Sweden; 5 UCL Institute of Neurology, Queen Square, London WC1N 3BG, United Kingdom; University of Hawaii, UNITED STATES

## Abstract

**Objective:**

To investigate the role of homocysteine in neuronal injury in HIV infection.

**Methods:**

Using a cross-sectional design and archived samples, we compared concentrations of plasma homocysteine and cerebrospinal fluid (CSF) neurofilament light protein (NFL), a sensitive marker of neuronal injury, in 83 HIV-1-infected subjects without antiretroviral treatment. We also analyzed plasma vitamin B_12_, serum folate, CSF, and plasma HIV RNA, the immune activation marker neopterin in CSF and serum, and albumin ratio as a marker of blood-brain barrier integrity. Twenty-two subjects provided a second sample median of 12.5 months after antiretroviral treatment initiation.

**Results:**

A significant correlation was found between plasma homocysteine and CSF NFL concentrations in untreated individuals (r = 0.52, *p* < 0.0001). As expected, there was a significant inverse correlation between homocysteine and B_12_ (r = –0.41, *p* < 0.001) and folate (r = –0.40, *p* = < 0.001) levels. In a multiple linear regression analysis homocysteine stood out as an independent predictor of CSF NFL in HIV-1-infected individuals. The correlation of plasma homocysteine and CSF NFL was also present in the group receiving antiretroviral therapy (r = 0.51, *p* = 0.016).

**Conclusion:**

A correlation between plasma homocysteine and axonal injury, as measured by CSF NFL, was found in both untreated and treated HIV. While this study is not able to prove a causal link, homocysteine and functional B_12_/folate deficiency appear to play a role in neural injury in HIV-infected individuals.

## Introduction

Prior to the discovery of effective antiretroviral therapy (ART), HIV-infected individuals had about a 30% overall risk of developing HIV-related dementia (HAD) [1]. Although the picture today is different, HIV diagnosis is to some extent still associated with HIV-associated neurocognitive disorders (HAND). Symptoms of HAND are common in untreated HIV-positive individuals and frequently reported by individuals on suppressive ART [2]. Recent studies have noted increased levels of cerebrospinal fluid (CSF) neurofilament light protein (NFL) in HIV-infected individuals, which is interpreted as a sign of ongoing neuronal injury. Elevated levels of CSF NFL have not only been reported in individuals with neurological symptoms, but also in some individuals with and without ART who are asymptomatic with regard to neurological symptoms [3,4].

**Fig 1:**
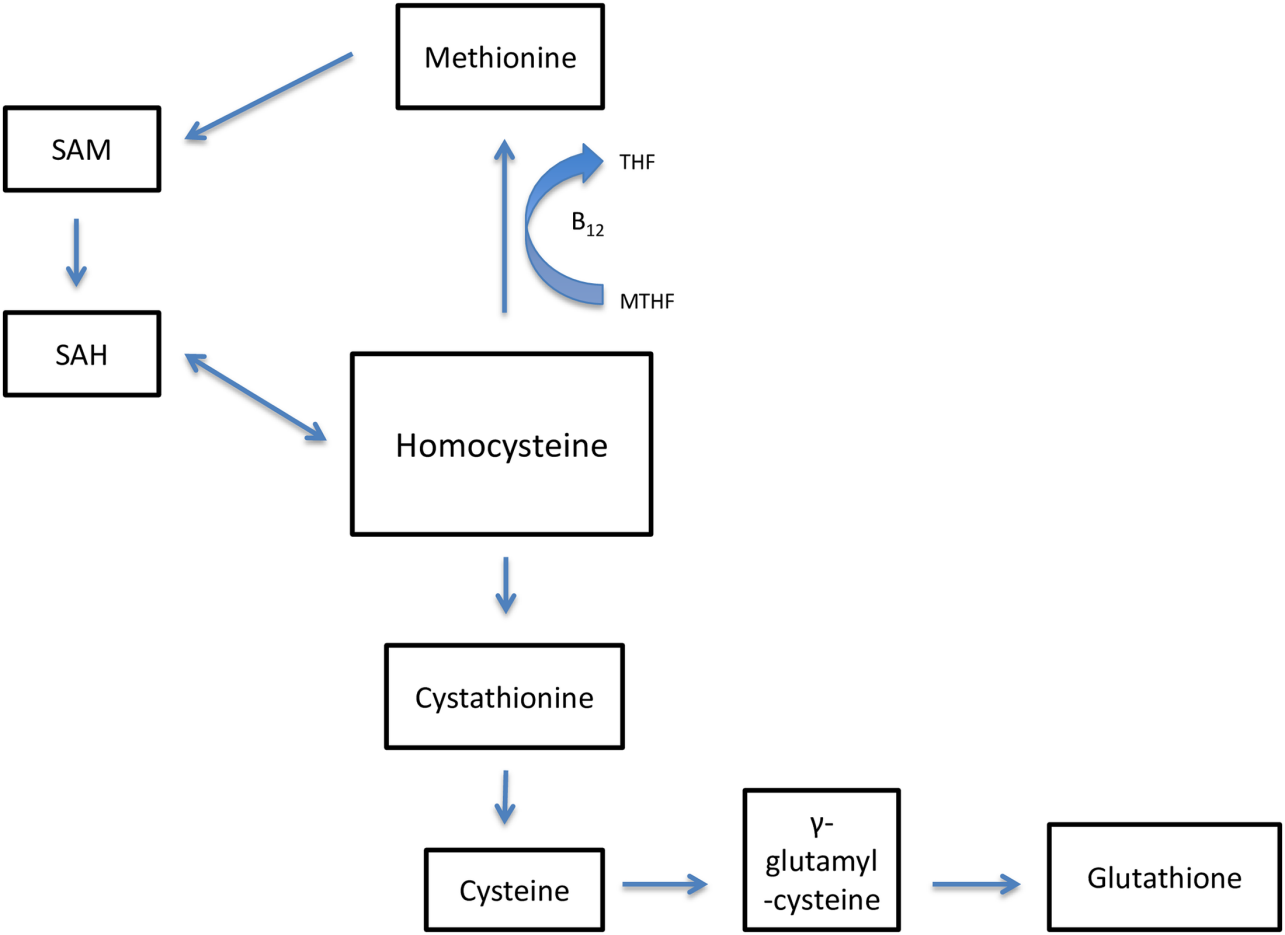
Homocysteine metabolism. Abbreviations: SAM, S-adenosyl-methionine; SAH, S-adenosyl-homocysteine; MTHF, methylenetetrahydrofolate; THF, tetrahydrofolate.

Associations between elevated plasma homocysteine levels and cognitive impairment in HIV-negative individuals have been the topic of many studies. Data suggest an association between elevated levels of homocysteine and diseases of cognitive impairment such as Alzheimer’s [5,6]. However, in the case of HIV-infected individuals, data on homocysteine levels in the context of CNS injury and neurocognition are rare. The metabolism of homocysteine is dependent on folate and vitamins B_12_ and B_6_, and therefore elevated levels of homocysteine are an indicator of B_12_ and/or folate deficiency (Fig 1) [7]. This study investigates the possible association between homocysteine and neuronal injury in HIV-1-infected individuals.

## Methods

### Study design

A retrospective selection was made of samples collected in a prospective research program, as previously described [8]. All samples were collected between 1999 and 2014 at the Department of Infectious Diseases at Sahlgrenska University Hospital, Gothenburg, Sweden. Study participants were randomly chosen from individuals in the cohort who met the inclusion criteria, i.e. untreated HIV-infection, over 18 years old, and no opportunistic CNS complications. Plasma and CSF specimens from 83 HIV-infected untreated individuals were analyzed (Table 1). Another plasma and CSF sample was obtained at a median of 12 months (range 10.8 to 27.1) in 22 subjects on ART. Subjects without signs of neurological or cognitive impairment at clinical examination were defined as neuroasymptomatic. Fifty-three of those had an asymptomatic HIV-infection (Center for Disease Control and Prevention [CDC] classification A1-A3) [9]. Twenty-two patients fulfilled the criteria for AIDS (CDC classification C2-C3), 13 due to pneumocystis pneumonia (PCP), four with tuberculosis, two with wasting syndrome, and one each with Kaposi’s sarcoma, extracerebral lymphoma, and salmonella sepsis. Five patients had symptomatic HIV-infections not categorized as AIDS (CDC classification B2-B3), all due to oral candidiasis.

**Table 1:** Characteristics of Study Population.

Group	N	Age	Gender	Plasma HIV RNA	CSF HIV RNA	CD4+ T-cells (x10e6)
		Median years (IQR)	M/F	Median Log10 (IQR)	Median Log10 (IQR)	Median (IQR)
**Total (untreated, NA)**	80	40.0 (33.0–49.0)	44/36	4.99 (4.32–5.47)	3.78 (3.10–4.32)	200 (60–360.5)
CD4 > 350	21	44.0 (31.0–52.0)	13/8	4.14 (3.60–4.76)	3.22 (2.29–3.92)	480 (400–670)
CD4 200–349	20	38.5 (31.75–43.75)	13/7	4.85 (4.32–5.35)	4.10 (3.37–4.47)	240 (218–275)
CD4 50–199	22	39.5 (34.25–52.5)	9/13	5.32 (4.75–5.64)	4.21 (3.81–4.75)	110 (75–148)
CD4 < 50	17	45.0 (38.0–47.0)	9/8	5.66 (5.16–5.91)	3.26 (2.23–3.75)	20 (10–30)
**HAD**	3	48.0 (42.0–52.5)	3/0	5.41 (5.18–5.87)	5.31 (4.81–5.36)	138 (99–184)
**ART**	22	43.5 (35.25–52.5)	10/12	< 1.7	< 1.7	395 (250–545)

Abbreviations: NA, neuroasymptomatic; HAD, HIV associated dementia; ART, Antiretroviral therapy

The untreated neuroasymptomatic individuals were divided into four subgroups according to CD4^+^ T-cell count (T-cells/μL): CD4^+^ > 350 (n = 21); CD4^+^ 200–349 (n = 20); CD4^+^ 50–199 (n = 22); and CD4^+^ < 50 (n = 17), based on earlier findings of higher CSF-NFL levels in untreated subjects with lower CD4 cell counts [3]. Three additional patients with HAD were tested but not included in the statistical analyses. All predictors were selected based on earlier findings of relevance to CNS HIV infection [3,10].

### Laboratory assays

All samples were frozen to –70°C within an hour of sampling and stored until analysis. CSF samples were centrifuged to remove cells and aliquoted before freezing. Levels of NFL in CSF were determined using a commercial ELISA, according to the manufacturer’s instructions (Uman Diagnostics, Umeå, Sweden). For neopterin a commercially available immunoassay (BRAHMS, Hennigsdorf, Germany) was used. CD4^+^ count, CSF, and blood albumin levels were analyzed according to local laboratory standards. CSF:blood albumin ratio was calculated as a measure of blood-brain barrier dysfunction.

Plasma vitamin B_12_ and serum folate were quantified using an electrochemiluminescence immunoassay on a cobas e analyzer (Roche, Penzberg, Germany). Total homocysteine was determined in EDTA plasma by stable isotope dilution liquid chromatography tandem mass spectrometry (LC-MS/MS) using a Quattro micro instrument (Waters Corporation, Milford, MA, USA), essentially as described by Magera et al. [11]. Levels of HIV RNA, both in CSF and plasma, were determined using the Roche Amplicor version 1.5, or Roche COBAS TaqMan assay version 1 or 2 (Hoffman La-Roche, Basel, Switzerland)

### Statistical analysis

Correlations were analyzed by Pearson correlation analysis. Paired T-test was used to compare the treatment group before and during treatment. Differences between groups were analyzed with ordinary one-way ANOVA and *P* values were adjusted for multiple comparisons using the Tukey’s Range test. Predictors of log_10_ plasma homocysteine and CSF NFL were analyzed by multiple linear regression analysis with forward selection. In the analyses CD4^+^ count, CSF:blood albumin ratio, plasma and CSF HIV RNA, serum and CSF neopterin, CSF NFL, plasma homocysteine, plasma vitamin B_12_, and serum folate were log transformed to reduce skewness. *P* < 0.05 was considered significant.

All statistical tests were performed with SPSS version 21 (IBM SPSS Statistics, Armonk, NY, USA) or Prism version 6.0 (Graphpad Software Inc., La Jolla, CA, USA).

### Ethics approval and informed consent

The entire study and the procedure used to assure the anonymity of the data was approved by the Research Ethics Committee of the University of Gothenburg, in accordance with the Helsinki Declaration of 1975, as revised in 2000. All participants gave their informed consent in writing. All patient information was anonymized prior to analysis by study Principal investigator (MG) at the Department of Infectious Diseases, at Sahlgrenska University Hospital.

## Results

### Descriptive statistics

Among the 80 untreated neuroasymptomatic subjects, 20 had moderate hyperhomocysteinemia (> 15 μmol/L). Twenty had elevated levels of CSF NFL, compared to age-dependent laboratory norms. B_12_ levels were below 140 pmol/L in 2 subjects, and below 250 pmol/L in 32. One individual had a high serum folate concentration. Of the three individuals with HAD, two had hyperhomocysteinemia and two had CD4^+^ counts < 200. All three had very high levels of CSF HIV RNA and CSF NFL.

### Group comparisons

**Fig 2:**
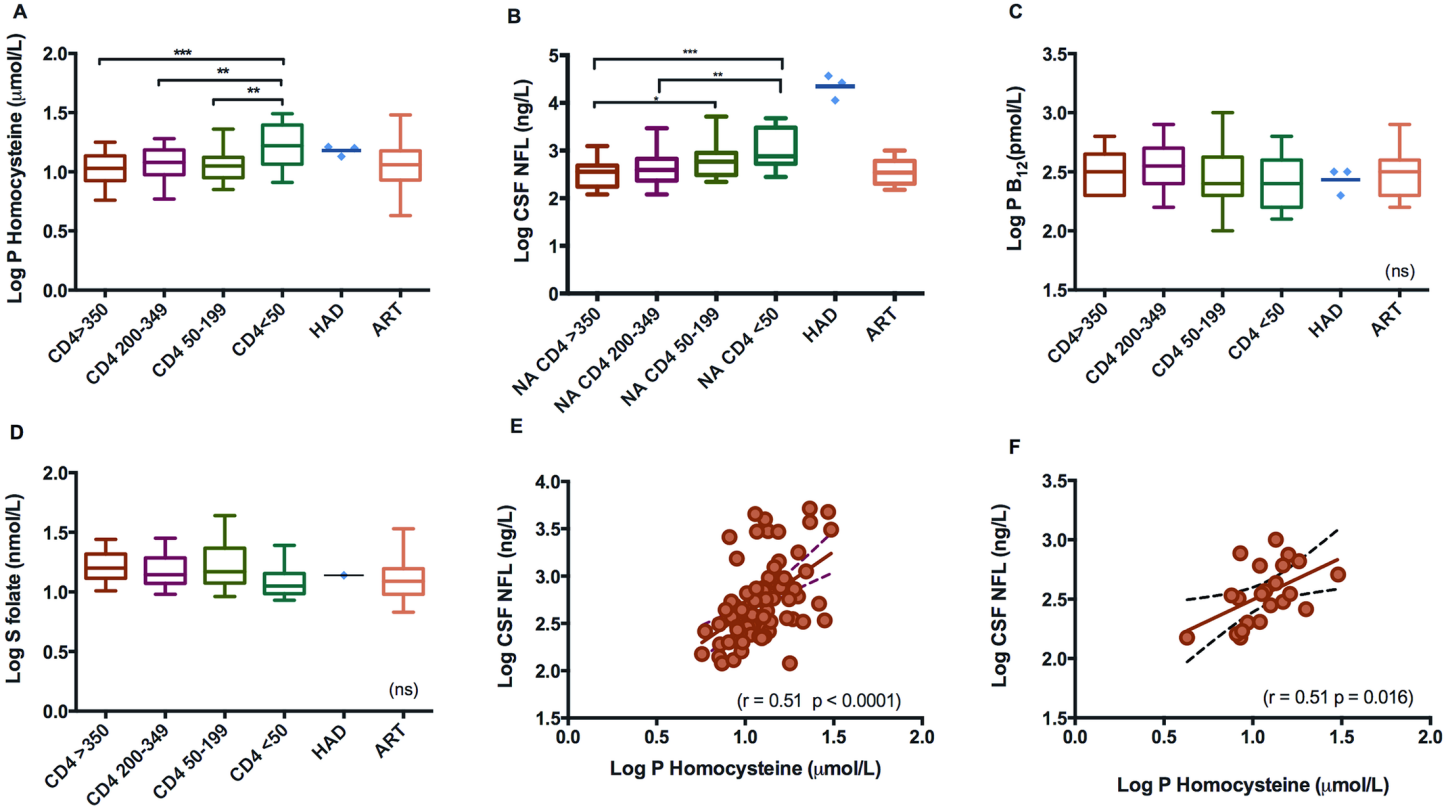
A-F. Levels of homocysteine, NFL, vitamin B12, and folate. Correlations of homocysteine and CSF NFL. A-D: Levels of homocysteine, NFL, vitamin B12, and folate in a cohort of HIV-infected individuals divided into groups. Neuroasymptomatic individuals are divided according to CD4+ T-Cell levels. Individuals with HAD and those on ART are presented as individual groups. * Adjusted *p* values. E: Correlation of Log P homocysteine and Log CSF NFL in 80 untreated, neuroasymptomatic HIV-infected individuals. F: Correlation of Log P homocysteine and Log CSF NFL in 22 neuroasymptomatic HIV-infected individuals on ART. Abbreviations: P, plasma; HAD, HIV associated dementia; ART, antiretroviral therapy; CSF, cerebrospinal fluid; NFL, neurofilament light protein.

Comparison of the subgroups of untreated neuroasymptomatic subjects showed a significant difference in homocysteine levels between the groups (*p* = 0.0002). The group with CD4^+^ count < 50 had homocysteine levels significantly higher than the other groups (Fig 2a). Differences between groups were also detected in levels of CSF NFL (*p* = 0.0001). The CD4^+^ < 50 group had significantly higher levels than the groups with CD4^+^ > 350 and CD4^+^ 200–349. The CD4^+^ 50–199 group had higher levels compared to the CD4^+^ > 350 group (Fig 2b). No significant difference between groups was found in plasma B_12_ or serum folate levels (Fig 2c and 2d).

### Predictors of CSF NFL and homocysteine

We found a significant correlation (r = 0.51, *p* < 0.0001) between the plasma level of homocysteine and CSF NFL (Fig 2e). As expected, there was also a significant inverse correlation of homocysteine and B_12_ (r = –0.36, *p* = 0.0009), and of homocysteine and folate (r = –0.40, *p* = 0.0003). Homocysteine was also found to correlate significantly to the CSF:blood albumin ratio (r = 0.25, *p* = 0.023), CD4^+^ count (r = –0.41, *p* = 0.0002), age (r = 0.40, *p* = 0.0002), and treatment with trimethoprim-sulfamethoxazole (r = 0.355, *p* = 0.001). Plasma homocysteine correlated to serum neopterin (r = 0.34, *p* = 0.002), but no correlation was found between homocysteine and CSF neopterin. No significant correlation was found between CSF NFL and B_12_ concentrations. A weak correlation was found between serum folate and CSF NFL (r = 0.28, *p* = 0.012).

Using multiple linear regression analysis age, plasma vitamin B_12_, serum folate, plasma HIV RNA, and treatment with trimethoprim-sulfamethoxazole were found to predict plasma homocysteine; however, serum neopterin and CD4^+^ count did not (Table 2). Plasma homocysteine, CSF neopterin, age, plasma HIV RNA, and CD4^+^ count stood out as independent predictors of CSF NFL, with adjusted estimates in the multivariate analysis. By contrast, CSF:blood albumin ratio, CSF viral load, serum neopterin, plasma vitamin B_12_, and serum folate were not found to be significant predictors (Table 3).

**Table 2:** Predicting Log P Homocysteine.

	Univariable		Multivariable	
Predictor	Std b (r)	*p*	Std b_adj_	*p*
Age	0.403	< 0.001	0.310	0.001
Log CD4	–0.411	< 0.001		
Log P HIV RNA	0.395	< 0.001	0.262	0.008
Log S Neopterin	0.340	0.002		
Log P B12	–0.363	0.001	–0.256	0.007
Log S Folate	–0.401	< 0.001	–0.191	0.039
Trimethoprim	0.355	0.001	0.225	0.020

Univariable correlation (left columns) and multiple linear regression (right columns) determining predictors of log10 plasma homocysteine in 80 HIV-infected neuroasymptomatic individuals without ART.

**Table 3:** Predicting Log CSF NFL.

	Univariable		Multivariable	
Predictor	Std b (r)	*p*	Std b_adj_	*p*
Age	0.475	< 0.001	0.327	0.001
Log CD4	–0.469	< 0.001	–0.287	0.003
Log CSF HIV RNA	0.058	0.609		
Log CSF Neopterin	0.379	0.001	0.271	0.002
Log CSF/P albumin ratio	0.267	0.016		
Log P B12	-0.165	0.143		
Log S Folate	-0.284	0.012		
Log P Homocysteine	0.507	<0.001	0.211	0.037

Univariable correlation (left columns) and multiple linear regression (right columns) determining predictors of log10 CSF NFL in 80 HIV-infected neuroasymptomatic individuals without ART.

### CSF NFL and homocysteine correlation after initiation of ART

The correlation of plasma homocysteine and CSF NFL was still present in the group on ART (Fig 2f). No significant differences in levels of plasma homocysteine or CSF NFL were found in the ART group before compared to during ART.

## Discussion

Our results show an independent correlation of plasma homocysteine and CSF NFL concentrations in neuroasymptomatic HIV-infected individuals, both untreated and those on ART. To the best of our knowledge, this is the first report that shows an association in any disease between NFL, a sensitive biomarker of neuronal injury, and plasma homocysteine.

Earlier studies have shown the prevalence of hyperhomocysteinemia in untreated HIV-infected individuals to be between 20% and 35% [12,13]. This is similar to our finding of 25%. Homocysteine levels increase with B_12_ vitamin and folate deficiency and are also influenced by renal function, age, and treatment with certain medications, e.g. trimethoprim. None of the subjects in our study had impaired renal function, and retrospective analysis of medical records showed that none were taking B vitamins. However, we discovered elevated levels of vitamin B_12_ in plasma in 14 samples, suggesting the possibility of B_12_ vitamin supplementation that was not recorded in patient files.

Trimethoprim treatment increased levels of homocysteine in a small number of healthy men [14]. However, no changes in homocysteine were seen in HIV-positive individuals on prophylactic doses of trimethoprim-sulfamethoxazole [15]. Seven individuals in our cohort received prophylactic and 13 treatment doses of trimethoprim-sulfamethoxazole. The correlation found between homocysteine and CD4^+^ cell count in the univariate analysis disappeared in the multivariate analysis. This may in part be explained by trimethoprim-sulfamethoxazole treatment of subjects with pneumocystis jiroveci pneumonia and low CD4^+^ cell count.

Elevated levels of CSF NFL are nearly always present in individuals with HAD [3,4], and increased levels can already be found 1 to 2 years before the development of overt dementia symptoms [8]. Consequently CSF NFL has been proposed as a predictive marker of HIV-associated neurocognitive disease before clinical symptoms appear [4,8]. In untreated neuroasymptomatic, HIV-infected individuals, markedly elevated CSF NFL levels signifying ongoing axonal injury are mainly found in those with low CD4^+^ cell counts [3]. Although our findings do not prove a causal link, they suggest homocysteine and functional B_12_/folate deficiency may be involved in the pathogenesis of CNS neural injury in HIV infection. In agreement with earlier findings, CSF NFL was higher in subjects with low CD4^+^ cells. CSF NFL also correlated with age and CSF neopterin. Moreover, as expected, correlations existed between age, B_12_, folate, and homocysteine.

HIV-infected individuals have increased levels of neopterin in CSF and serum as a result of immune activation [10]. Earlier studies of HIV-negative individuals have found a correlation between homocysteine and serum neopterin, implying an association between homocysteine and immune activation [16]. It has been hypothesized that neopterin, through the influence on folate metabolism, inhibits methionine synthase [17]. In addition, nitric oxide from activated macrophages may also inhibit the enzyme [18]. Another proposed explanation of the role of immune activation in hyperhomocysteinemia is through the oxidation of B_12_ and folate. This could give rise to a functional deficiency that has the potential of both enhancing oxidative stress and perturbing the methylation process [19]. However, we did not find serum neopterin to be an independent predictor of homocysteine in multivariate analysis. In an earlier study of individuals with dementia, treatment with B vitamins reduced the level of homocysteine but did not influence neopterin concentrations [20].

The measurement of B_12_ is a poor marker of B_12_ deficiency, whereas homocysteine is more sensitive [21]. This may partly explain the lack of correlation we found between CSF NFL and plasma vitamin B_12_. The inhibition of crucial enzymes, or the lack of adequate forms of B_12_/folate, may also explain disturbances in the homocysteine metabolism, even when there are sufficient levels of B_12_ and folate. The homocysteine lowering effect of B vitamin treatment supports the presence of functional B_12_ and/or folate deficiency in the HIV-negative population. The limited data available indicates that this is also the case in HIV-positive individuals [22].

Significantly, the correlation between CSF NFL and plasma homocysteine remained present in the group on ART. We found a correlation between homocysteine and HIV RNA levels in serum in untreated HIV, but we did not find a significant change in homocysteine levels before compared to after ART initiation.

Previous studies of the association of B vitamin levels and neurocognitive function in untreated HIV-infected individuals have been inconclusive [23–25]. Impaired information processing speed and visuospatial problem solving abilities have been reported in untreated, neuroasymptomatic HIV-infected individuals with B_12_ deficiencies [23]. A small study reported improvement of neuropathic symptoms in such individuals when given replacement therapy [24].

There are several limitations to our pilot study: it is of retrospective design; the sample size was limited; no HIV-negative control group was included; and neuropsychiatric testing was not performed on all asymptomatic subjects.

## Conclusion

We found a correlation between plasma homocysteine and axonal injury, as measured by CSF NFL, in both untreated and treated individuals with HIV. While our study is unable to prove a causal link, homocysteine and functional B_12_/folate deficiency appear to play a role in neural injury in HIV-infected individuals that is worth exploring.

## Supporting Information

S1 Table Full set of the data included in the analysis. (XLSX)
